# Supplementary material for: Emotion regulation and compassion fatigue in mental health professionals in a context of stress: A longitudinal study
Source: PLOS Ment Health. 2025 Feb 19;2(2):e0000187. doi: 10.1371/journal.pmen.0000187 (PMC12798466; doi:10.1371/journal.pmen.0000187)
Supplement: S1 Table — (DOCX) [file pmen.0000187.s001.docx]

**S1 Table.**

*Correlation Matrix of Study Variables*

| **Variables** | *M* | *SD* | 1 | 2 | 3 | 4 | 5 | 6 | 7 | 8 | 9 | 10 | 11 | 12 | 13 | 14 |
| --- | --- | --- | --- | --- | --- | --- | --- | --- | --- | --- | --- | --- | --- | --- | --- | --- |
| 1. Age | 42.32 | 11.35 | -- |  |  |  |  |  |  |  |  |  |  |  |  |  |
| 1. Sex | 0.11 | 0.31 | .20** | -- |  |  |  |  |  |  |  |  |  |  |  |  |
| 1. Work modalities | 0.59 | 0.49 | 0.04 | -0.06 | -- |  |  |  |  |  |  |  |  |  |  |  |
| 1. Changes in workload | 0.30 | 0.46 | -0.02 | -0.02 | -.14** | -- |  |  |  |  |  |  |  |  |  |  |
| 1. T1 Physical stressors | 0.22 | 0.53 | .24** | .11* | -0.01 | 0.05 | -- |  |  |  |  |  |  |  |  |  |
| 1. T1 Contextual stressors | 1.52 | 1.27 | .11* | -0.07 | 0.09 | 0.02 | .20** | -- |  |  |  |  |  |  |  |  |
| 1. T1 Perceived stress | 4.07 | 1.30 | -0.01 | 0 | 0.02 | 0.02 | .24** | .20** | -- |  |  |  |  |  |  |  |
| 1. T1 Dysregulation | 3.25 | 1.08 | -.18** | -.11* | -0.01 | 0.05 | 0 | 0 | .26** | -- |  |  |  |  |  |  |
| 1. T1 Integration | 5.73 | 0.87 | 0.04 | -.16** | 0.09 | 0.05 | -0.09 | 0.1 | -0.05 | -.10* | -- |  |  |  |  |  |
| 1. T1 Suppression | 2.99 | 1.21 | -.10* | 0.07 | -.17** | 0.02 | 0.07 | -0.05 | 0.05 | .27** | -.26** | -- |  |  |  |  |
| 1. T1 Compassion fatigue | 2.55 | 0.44 | -0.03 | -.12* | -.13** | .18** | 0.02 | 0.07 | .17** | .36** | -0.01 | .21** | -- |  |  |  |
| 1. T2 Dysregulation | 3.17 | 1.11 | -.22** | -.11* | 0.03 | -0.01 | 0.01 | 0.01 | .25** | .66** | -.10* | .19** | .30** | -- |  |  |
| 1. T2 Integration | 5.72 | 0.83 | 0.05 | -.13** | .10* | -0.07 | -0.03 | 0.02 | 0 | -.15** | .55** | -.31** | -.12* | -0.08 | -- |  |
| 1. T2 Suppression | 3.09 | 1.17 | -0.07 | 0.02 | -.15** | 0.07 | 0.07 | -0.02 | 0.08 | .16** | -.20** | .58** | .15** | .21** | -.37** | -- |
| 1. T2 Compassion fatigue | 2.67 | 0.46 | -0.04 | -0.09 | -0.05 | 0.05 | -0.01 | 0.07 | .16** | .32** | -0.03 | .12* | .58** | .35** | -.14** | .20** |

*Note.* T1 = Time 1; T2 = Time 2. **p*<.05, ** *p* <.01.
