# Supplementary material for: Emotion regulation and compassion fatigue in mental health professionals in a context of stress: A longitudinal study
Source: PLOS Ment Health. 2025 Feb 19;2(2):e0000187. doi: 10.1371/journal.pmen.0000187 (PMC12798466; doi:10.1371/journal.pmen.0000187)
Supplement: S2 Table — (DOCX) [file pmen.0000187.s002.docx]

**S2 Table.**

*Fit of the multigroup analyses for the two moderators*

| Variable | Groups | χ^2^ | | | CFI | TLI | RMSEA | | | SRMR |
| --- | --- | --- | --- | --- | --- | --- | --- | --- | --- | --- |
|  |  | Value | *df* | *p value* |  |  | Value | LB 90% CI | HB 90% CI |  |
| Saturated model | *–* | 0.00 | 0 | 1.00 | 1.00 | 1.00 | .000 | .000 | .000 | .000 |
| Criteria for equal fit | *–* | *–* | *–* | >.05 | ± 0.01 | ± 0.01 | ± .015 | *–* | *–* | *–* |
| Work modalities | In person: n= 162  Telepractice: n= 228 | 3.19 | 6 | .79 | 1.000 | 1.000 | .000 | .000 | .061 | .012 |
| Change in workload | Same or reduced: n= 273  Increased: n= 117 | 5.57 | 6 | .47 | 1.000 | 1.000 | .000 | .000 | .089 | .015 |

Note. χ² = chi-square model fit; df = degrees of freedom; CFI = comparative fit index; TLI = Tucker-Lewis index; RMSEA = root mean square error of approximation; CI = confidence interval; LB = lower bound; HB = higher bound; SRMR = standardized root-mean-square residual.
