# Supplementary material for: Emotion regulation and compassion fatigue in mental health professionals in a context of stress: A longitudinal study
Source: PLOS Ment Health. 2025 Feb 19;2(2):e0000187. doi: 10.1371/journal.pmen.0000187 (PMC12798466; doi:10.1371/journal.pmen.0000187)
Supplement: Attachment — Submitted filename: Response to Reviewers.docx [file pmen.0000187.s003.docx]

October 28, 2024

PMEN-D-24-00323
Stressors Change Emotion Regulation in Mental Health Professionals Over Time: Consequences for Depression and Compassion Fatigue
PLOS Mental Health

Dear Dr. Gareth Hagger-Johnson,

Thank you for your decision letter of August 22, 2024 recommending that we revise our manuscript initially titled “Stressors Change Emotion Regulation in Mental Health Professionals Over Time: Consequences for Depression and Compassion Fatigue”. We have carefully considered all your comments and those of the reviewers and addressed all of them in the current letter and made the appropriate changes where needed. We have included an unmarked and a marked-up copy of our manuscript highlighting the changes made to the original version.

**Editor’s comments**

**1. The analysis is far too complicated, and should be simplified. The research question is not precise enough, which gives the paper a very exploratory feel. I think the paper covers too much ground. The study was not pre-registered, compounding this feeling further. PLOS recommends that studies and research question are pre-registered where possible.** [**https://plos.org/open-science/preregistration/**](https://plos.org/open-science/preregistration/)

We agree that the research question was not precise enough. We now focus on the bidirectional relationship between emotion regulation and compassion fatigue in a context of stress. We have now also clarified the hypotheses relative to this model. Moreover, we have tremendously simplified the analysis and the model. We detail that simplification below in responding to your other related comments.

**2. In the text, the status of covariates/confounders, mediators, effect modifiers (moderators) and outcomes is sometimes obscure (see https://doi.org/10.1093/ije/dyi074). Effect modification is not typically used to test ‘robustness’, it is usually driven by a priori hypotheses about why a predictor or risk factor might have different effects for different groups (‘different slopes for different folks’). Papers usually consider one or two effect modifiers maximum (e.g. age, sex), not four, and I’m not convinced multiple group SEM is the way to do this. Moderated mediation analysis is a possible alternative, but might make things even more complex.**

We agree that we examined too many potential moderators. We now only examine two that are related to the nature of our sample composed of mental health professionals: work modality and workload. This also takes care of the issue of dichotomizing continuous moderators at random cut off points (e.g., at the mean) since work modality is binary and changes in workload examines reduced or same amount of workload vs. an increase amount. To examine these moderations, we kept using multiple group SEM analyses since this allowed for moderated mediations in our previous model. However, to reduce the complexity of the model and to clarify the research question, we now only present a general cross-lagged model between emotion regulation and compassion fatigue, with the addition of perceived stress modeled as a predictor of both emotion regulation and compassion fatigue at Times 1 and 2. The research question is now clearly focused on the direction of the effect between emotion regulation and compassion fatigue in a context of stress. We kept the other variables as covariates in the model (i.e., age, sex, work modality, workload).

**3. You controlled for age (e.g. Table 1) but not other covariates. I recommend testing for effect modification and if there is no statistical evidence for this, age, sex, work modality etc. should be treated simply as covariates. Include them all in the model and in Table 1, but no need to show them in the path diagram. I had to read the text, figure and table several times in order to work out that status of each variable, which means readers would too, so it needs rewriting to help readers understand clearly what is happening without having to re-read. However, I think this can all be addressed with clearer language, careful introduction of the status of each variable, and a simplified model.**

As per your above recommendation, we now test for two moderators only that are related to the type of sample we used (mental health professionals working during the pandemic). We have now included all covariates in the model (age, sex, work modality, and workload) and their standardized path coefficients can be found in Table 1.

**4. You did not measure a key confounding factor (trait neuroticism) which could be driving most of the covariance over time. It is likely that participants low in a general tendency to experience negative emotions were buffered. It is an unmeasured confounder and an important limitation.**

We agree that it is a limitation that we did not measure neuroticism, and we now acknowledge this in the limitation section (see p. 19). However, the research question is about the direction of the effect between emotion regulation and compassion fatigue. We therefore look at the changes in emotion regulation and compassion fatigue over time, while controlling for their respective baseline. We believe there is sufficient evidence showing that neuroticism is a distinct construct from emotion regulation and compassion fatigue (e.g., Korkmaz & Gökoğlan, 2024; Pont et al., 2020) to presuppose that the effect is not entirely driven by neuroticism.

**5. Another limitation is that there are only two timepoints. With three, latent growth curve modelling would have provided a flexible way to handle individual differences and genuine change over time, e.g. parallel process growth models. Ease of interpretation is further complicated by referring to ‘change’ (p.7) in a variable as a mediator. The meaning of ‘change’ is obscure. Is this time 2 value with baseline adjustment for time 2? Or simply the value at time 2? Or the difference between time 1 and time 2?**

We have now simplified our model to a general cross-lagged model. This clarifies the notion of change in our model, which implies a residualized change score in the variance of the variables from T1 to T2, not their mean score (intercept). It is true that three time points would have provided more information on the nature of the change, either its intercept or slope. We have added this as a limitation (p. 19). Of note, we do not assess mediation anymore, which clarifies the model and the meaning of change in that context.

**6. When you only have two timepoints, the meaning of change is not straightforward (see for example https://academic.oup.com/ije/article/51/5/1604/6294759 and the Glymour paper they mention). I think this would be more easily dealt with by focusing on one outcome separately in linear regressions, with cumulative exposure scores for predictors at time 1 and 2 (see below).**

By change we imply predicting Time 2 variable while adjusting it for its Time 1 measure. This is a residualized change score, not a mean difference score (T2 – T1). Note that, as adequately pointed out by Tennant et al., 2021, using either the full score of Time 2 variable or a difference score (T2 – T1) is mathematically equivalent when controlling for Time 1 variable in the model. Tennant et al., 2021 suggest that it is the only way to compute true score change and this is what we did. Note, however, that in a saturated cross-lagged model, with two Time 1 variables (e.g., X1 and Y1) and two Time 2 variables (X2 and Y2), the parameter estimates of the Time 1 exogenous variables (X1 and Y1) on T2 outcomes will not change whether there is one or two Time 2 variables (only X2 or only Y2 vs. X2 and Y2). This is because path analyses estimate them separately and only their T2 measurement errors covary in the model. Thus, removing one T2 variable (e.g., Y2) and preserving all T1 variables as covariates (X1 and Y1) does not change at all the results. This is mathematically equivalent to a regression analysis with only one dependent variable. This means that a cross-lagged model with two measurement timepoints is mathematically equivalent to a linear regression predicting T2 variable while adjusting for its T1 variable (as advised by Tennant et al, 2021) or an ANCOVA with one T2 variable as the dependent variable and its relative T1 variable as the covariate (ANCOVA is just a special case of regression). For this reason, we have kept the cross-lagged model (in a much-simplified version) and we have clarified what we meant by change in the manuscript (see p. 11). **Also see point 8 below on why we did not use other types of analyses with two time points.**

**7. If the research question is ‘does emotion regulation cause psychopathology or vice versa’ (this was clearer on p. 21) then please refocus the paper on that question only, keeping the model as simple as possible.**

We have now refocused the paper on whether emotion regulation affects compassion fatigue or the other way around. We have also removed from the model the physical and contextual factors predicting perceived stress. We know only show that they are related to perceived stress in the descriptive analyses.

**8. Unless there is strong evidence for effect modification, no need to do multiple group analysis, just state where it was not found. Adjust for covariates but show the X, M, Y only at each timepoint in a simplified figure. If you really want to use path models, consider the random-effects cross-lagged panel model (RE-CLPM e.g. https://doi.org/10.3758/s13428-017-0979-2) clearly specifying which is your predictor, mediator and outcome. Regress everything on age, sex, work modality etc. but don’t show these in the path diagram. Only separate groups if effects are materially different, and conclusions. Keep the path diagram as simple as possible (for example, Figure 1 in Deary et al. 2009, Psychology & Aging) and with wide readership in mind.**

Unfortunately, RE-CLPM is not identified with only two measurement time points, which means it just cannot be computed since there are more fixed parameters than the number of degrees of freedom afforded by the model. Therefore, we have used a standard CLPM model with the following modification to the model. First, we have removed the physical and contextual factors from the model to keep it simpler. Second, we have removed depression and kept compassion fatigue, since this latter variable is more relevant to mental health professionals, that depression and compassion fatigue are highly correlated, and that the results were exactly the same with either depression or compassion fatigue. This also allowed us to make our theoretical question simpler, by dropping the distinction between depression and compassion fatigue. Third, we have simplified our model and we now use a standard CLPM with all Time 1 variables predicting all Time 2 variables. We do not model changes in emotion regulation at Time 2 as predictors of changes in compassion fatigue at Time 2 anymore. Fourth, as per your recommendation, we have limited the number of moderators to two. We kept using multigroup analyses to test for their effect as this is what is recommended in that context (e.g., Memon et al., 2019). Since there was no significant moderation, we only mention it in the manuscript and we only control for these variables as covariates in the final model (in addition to age and sex) and we do not show them in the Figure.

**9. In summary, there are several predictors, several mediators, several effect modifiers, and several outcomes – this is all too complex. Please rewrite the introduction to identify a specific gap in the literature, and then introduce a simplified parsimonious model with one (or two) mediating pathways that would answer the question about the most likely causal / reverse causal direction of effects.**

We have rewritten the introduction to clarify the research question and the hypotheses. We now only focus on emotion regulation and compassion fatigue in a context of stress. We show that perceived stress increases emotion dysregulation over time and that there is a bi-directional effect between emotion dysregulation and compassion fatigue. We believe this is a clearer model that maps better with the research question.

**10. Note that where you have done multiple group analysis, dichotomising variables reduces statistical power and is usually not recommended. Another limitation is that it pre-supposes we know in advance where the appropriate cut point is, which will depend on whether the effect modification is linear or not. Some effects are threshold effects, only appearing in the top tertile or even deciles. Some effects are quadratic. The midpoint might not be the place to cut.**

Yes, we agree with this. We had done that since this allowed us to use multiple group SEM, which is computationally less intensive than modeling moderations with continuous variables among each variable of the model (especially considering that our model was quite complex, as you pointed out). We now only focus on work modality (telepractice vs. in person) and change in workload (same or reduced vs. more) as moderators. Reducing the number of moderators to two takes care of the dichotomizing problem and the random midpoint cut off. We control for age, sex, work modality, and workload as covariates in the main model.

**11. I have concerns about the possibility that those who dropped out at Time 2 differ significantly from those who remained in the study. To address this potential issue, a Bonferroni correction is insufficient in my opinion, because it ignores correlations among predictors which could mask important adjusted differences. Instead, I recommend using logistic regression then inverse probability weighting (IPW) to adjust for non-ignorable dropout. This method will help reduce bias in your main analysis.**

We have now used a logistic regression by regressing dropout status on all Time 1 variables. No variable was predictive of dropout status at p < .05. Therefore, we did not weight the cross-lagged model for dropout status. We now provide the non-significant result of the logistic regression in the manuscript (see p. 9).

**12. Please include a STROBE checklist and page numbers where each item in the checklist is addressed, in a revision**

We have included a STROBE checklist and the page number to locate each item.

**13. OTHER MINOR EDITS NEEDED**

**13.1 Cover page - Why isn’t the first author the corresponding author?**

In our field, the last author is often the corresponding author and the main researcher.

**13.2**

**Abstract – semicolon should be comma
P5 ‘carry over’ not precise enough
P5 Define key terms at first usage (‘Emotion regulation’)
P6 ‘buffer’ isn’t precise enough a term
P4 ‘depression transpires in all spheres of life’ – not clear what this means**

**P9 Extra space in 4,904 deaths. Both death figures need commas.**

**P23 Should be ‘begin to dysregulate’ not ‘begin to be dysregulated’**

All these instances have been corrected or deleted.

**13.3 P3 You need (at least in the discussion) to reflect on whether the 25% in private practice might be protected by the additional resources they may have.**

Private practice may or may not include more resources than practice in the public sector. At the opposite, in the public system, mental health professionals were more quickly provided with the tools and resources they needed during the pandemic (e.g., masks, sanitizer, offices with appropriate physical distance, videoconference licenses) than let’s say a psychologist working in a private office.

**13.4 P8 What proportion are men/women? Why is this missing? If they are mostly women, acknowledge in the discussion as a limitation, but do control for sex as a covariate. You seem to have sufficient numbers of each to test for multiple group analysis so I’m not clear why it’s not reported as a descriptive statistic here.**

Gender information was already provided in our manuscript (see p. 8). The sample consists of 89.5% of females, which accurately represents the proportion of females working as mental health professionals in Canada. Specifically, 80% of the general health sector workforce is female (<https://www150.statcan.gc.ca/t1/tbl1/fr/tv.action?pid=1410033502>), with 80.2% of psychologists, and 90% of social workers being women. This composition aligns with the actual demographics of the study population; therefore, we do not consider this a limitation. Howver, we did control for sex in the analyses.

**13.5 P9 There is an ethical approval statement here masked for review, then another ethical approval statement on page 10, unmasked. Please include one unmasked review (the review process is transparent anyway, no need to mask).**

We have unmasked the ethical statement and removed the duplicate mention.

**13.6 P11 How can past research be a paper published in 2022, for your study conducted in 2020/21?**

This paper pertains to a study also conducted during the pandemic in 2020, which uses the same scale, but which was used and validated with a sample from the general population. We have now clarified this information in the manuscript (p. 10).

**13.7 P24 Not controlling for trait negative effect (neuroticism) is a major limitation and should be acknowledged more strongly here. If you had found incremental validity beyond this trait, it would be a stronger argument to focus on these constructs more. I am not convinced yet that the findings aren’t driven by stable personality traits.**

See our response to this issue in the point #4 above.

**13.8 P14 A statement should be included that data and code will be deposited in a repository for open access (or the reason if not).**

We now provide such a statement on p. 12.

**Reviewer #1:** 

**1. It is important to recognise and be explicit about the practice context of the research participants, e,g, cultural, professional-clinical and regulatory factors may contribute and also affect application/generalisability of findings.**

All information regarding the practice context of the participants is provided on p.8. This includes, their level of education, gender, types of profession, work settings, patient population, and work modality.

**2. Modifying factors, such as requirements for the provision and uptake of clinical supervision, caseload/workload restrictions, vary significantly between professions, countries/state regulators of professions and providers of services, and in their evidence base. It would be insightful to understand if there is significant difference in reported outcomes when demographic and contextual factors are triangulated.**

As sensitivity analyses, we have examined whether work modality during the pandemic and changes in the workload had affected emotion regulation and compassion fatigue over time and whether these variables moderated the results obtained. Results show that these two variables did not have any direct or moderating effect.

**Reviewer #2:**

**1. This paper is strong in its reliability and replicability however may be reductive to individual experiences in that context and time. This does not reduce the effectiveness of the current paper at all as the goals of the paper are very clear. it meets the goals that it sets out well.**

We agree that our results can hardly be generalized with confidence to another stressful context. We have clarified this limitation in the manuscript. See p. 19.

**2. I really like how different professional backgrounds are highlighted and considered. Being a social worker in a mental health team myself, I often feel like a minority and my professional knowledge and experience can be reduced to silence. One of the limitations that the paper highlights was that there was not equal representation of professional groups. This is likely the case due to mental health teams being largely health and psychology lead.** **An interesting area for future study would be to explore more specifically the variances between different professional groups. Policies that we have had in the UK aimed at creating a more generic professional role in mental health such as “new ways of working” as well as the change from the Approved social worker (ASW) to the Approved Mental Health Practitioner (AMHP) erode professional identity. I’m not familiar with Canadian policies but it is well documented that in a workplace setting lead by a medical institution, some professional identities can be lost due to introduction of generic roles such as care-coordinator or allied health professional. For instance, Nathan and Webber (2010) argued that the specialised knowledge and skills of the social worker (social and environmental determinants of mental health) can be lost to a more bio-medical infrastructure. I would be interested to understand more how the loss of professional identity may impact job satisfaction which may in turn impact compassion fatigue and depression over time. My hypothesis would be that those with a non-medical background such as social work would be less resilient to longer term depression and compassion fatigue due to feeling less satisfied with their contribution and not having the same sense of value.**

This would be an interesting avenue for future research, but this research question is too different from the one we are addressing in the current paper. There are certainly several organizational factors contributing to compassion fatigue. We appreciated that this reviewer shared this interesting hypothesis with us.

**Another thing that stood out to me was that the overwhelming majority of the sample was female (89.5%). It would be interesting to have a more focused study into how stressors can implicate emotional regulation in male mental health practitioners, or to compare the variations between males and females in more detail. There may be differences t how they regulate or supress emotions based in socially expected behaviour on an individual level as well as wider societal and cultural norms.**

Yes, that would be interesting to have more males in our sample. Unfortunately, this represents the current proportion of males engaged in mental health professionals. See Editor’s points # 13.4 above.

**The paper discusses clinical implications and offers some examples of how work conditions may be addressed. One recommendation that I would make would be for government to adequately fund and provide additional resource to mental health teams, which would reduce stressors. A lot of emphasis is placed on the individual when conversations are had about burnout or compassion fatigue. Mental health services are chronically under funded. Mental health professionals are forever being told to take breaks, do self care, and get adequate supervision, but the diversion of the problem onto the individual can only go so far.**

This is a good and important point. We have added this idea in the General Discussion (see p. 19).

In sum, we have addressed all of your comments and those of the Reviewers and made the appropriate changes to the manuscript. We believe the manuscript is now much improved. We want to thank you all for your constructive comments and suggestions and look forward to hearing from you.

Sincerely,

Frederick L. Philippe, Ph.D.
Full Professor
[ELABORER | Laboratory for Research on Emotions and Representations](https://www.elaborer.org)
[UQAM Research Chair | Memory, Aversive Events, and Mental Health](https://recherche.uqam.ca/unites_de_recherche/chaire-de-recherche-uqam-sur-la-memoire-les-evenements-aversifs-et-la-sante-mentale/)
Department of psychology | University of Quebec at Montreal
[philippe.frederick@uqam.ca](mailto:philippe.frederick@uqam.ca)

Pavillon Adrien Pinard, SU-4385
100 Sherbrooke West
Montreal (QC), Canada H2X 3P2
Phone: (514) 987-3000 #5098 | Fax: (514) 987-7953
